# Association between *Helicobacter pylori* eradication and the risk of coronary heart diseases

**DOI:** 10.1371/journal.pone.0190219

**Published:** 2018-01-02

**Authors:** Jiunn-Wei Wang, Kuo-Lun Tseng, Chien-Ning Hsu, Chih-Ming Liang, Wei-Chen Tai, Ming-Kun Ku, Tsung-Hsing Hung, Lan-Ting Yuan, Seng-Howe Nguang, Shih-Cheng Yang, Cheng-Kun Wu, Chien-Hua Chiu, Kai-Lung Tsai, Meng-wei Chang, Chih-Fang Huang, Pin-I Hsu, Deng-Chyang Wu, Seng-Kee Chuah

**Affiliations:** 1 Division of Gastroenterology, Department of Internal Medicine, Kaohsiung Medical University Hospital, Kaohsiung Medical University, Kaohsiung, Taiwan; 2 Division of Gastroenterology, Kaohsiung Municipal Ta-Tung Hospital, Kaohsiung, Taiwan; 3 Division of Gastroenterology, Cishan Hospital, Kaohsiung, Taiwan; 4 Department of Pharmacy, Kaohsiung Chang Gung Memorial Hospital, Kaohsiung, Taiwan; 5 School of Pharmacy, Kaohsiung Medical University, Kaohsiung, Taiwan; 6 Division of Hepato-gastroenterology; Department of Internal Medicine, Kaohsiung Chang Gung Memorial Hospital, Kaohsiung, Taiwan; 7 Chang Gung University, College of Medicine, Kaohsiung, Taiwan; 8 Division of Gastroenterology, FooYin University Hospital, Pin-Tung, Taiwan; 9 Division of Hepato-gastroenterology; Department of Internal Medicine, Buddist Tzu Chi General Hospital, Dalin Branch, Chia-Yi, Taiwan; 10 Divisions of Gastroenterology, Yuan General Hospital, Kaohsiung, Taiwan; 11 Division of Gastroenterology, Ping-Tung Christian Hospital, Pin-Tung, Taiwan; 12 Division of Nephrology; Department of Internal Medicine, Kaohsiung Chang Gung Memorial Hospital, Kaohsiung, Taiwan; 13 Division of Colon and Rectal Surgery, Department of Surgery, Kaohsiung Chang Gung Memorial Hospital, Kaohsiung, Taiwan; 14 Departmemt of Emergency Medicine, Kaohsiung Chang Gung Memorial Hospital, Kaohsiung, Taiwan; 15 Division of Family physician, Kaohsiung Chang Gung Memorial Hospital, Kaohsiung, Taiwan; 16 Division of Gastroenterology, Department of Internal Medicine, Kaohsiung Veterans General Hospital, National Yang-Ming University, Kaohsiung, Taiwan; National Cancer Center, JAPAN

## Abstract

The evidences on the association of *Helicobacter pylori* (*H*. *pylori*) to coronary heart diseases (CHD) are conflicting. In order to answer this important but yet unanswered clinical health issue, a large cohort study such as big data from the Taiwan National Health Insurance Research Database should be more convincing. Therefore, we aimed to make use of these big data source to analyze and clarify the relevance of *H*. *pylori* eradication and CHD risks. We looked through a total of 208196 patients with peptic ulcer diseases (PUD) from the years of 2000 to 2011. First, 3713 patients who received *H*. *pylori* eradication within 365 days of the index date were defined as the group A. We randomly selected the same number of patients as cohort A from 55249 non-eradication patients to be the comparison group B using propensity scores (including age, gender and comorbidity) so that we could control the confounding variables of CHD and mortality. Importantly, we perform sensitivity analysis for the time-dependent association between *H*. *pylori* eradication and risk of CHD, interactions between patient demographic characteristics and therapy by age (≥ or < 65 years old). The results showed that a trend of decreased association of CHD in patients with early eradication was observed compared to those without eradication (2.58% vs. 3.35%, *p = 0*.*0905*). The mortality rate was lower in early eradication subgroup compared to cohort B (2.86% vs. 4.43%, *p = 0*.*0033*). Interestingly, there was also significant difference observed in composite end-points for CHD and death in the early eradication subgroup (0.16% vs.0.57%, *p = 0*.*0133*). Further, the cumulative CHD rate was significantly lower in younger patients (< 65 years old) with *H*. *pylori* eradication therapy started < 1 year compared to those patients without eradication at all (*p = 0*.*0384)*; the treatment did not appear to have an effect in older patients (≥ 65 years old) (*p = 0*.*1963*). Multivariate analysis showed that hypertension and renal diseases were risk factors for CHD in patients without eradication whilst younger age (< 65 years old) initiated with *H*. *pylori* therapy was a protective factor. In conclusion, the trend of decrease in CHD occurrence after early *H*. *pylori* eradication in addition to the significant decrease in composite end points for CHD and death, the significantly lower cumulative CHD rate in younger patients < 65 years old with *H*. *pylori* treated within 365 days suggested that there was positive association between *H*. *pylori* eradication and CHD.

## Introduction

Coronary heart disease (CHD) is the most common type of heart disease and characterized by atherosclerosis in the epicardial coronary arteries. Atherosclerosis is considered as a chronic inflammatory disease of blood vessels. Many studies suggested that infection with microbes and inflammation at the site of vessel wall have effects on the formation of atherosclerotic plaque and fasten the process of atherosclerosis [1,2]. In recent years, more and more evidences have come to the literature proposing association between CHD and infectious microbes, including those intracellular pathogens such as *Helicobacter pylori* (*H*. *pylori*) [3]. *H*. *pylori* infection relates to the development of gastrointestinal diseases and extra-gastrointestinal disorders [4–8]. The effects of *H*. *pylori* in the pathogenesis and prognosis of CHD still remained controversial. Some previous studies had shown a positive correlation between *H*. *pylori* infection and CHD, whereas others demonstrated that the correlation was only because of confounding effects [9–11]. Moreover, several meta-analyses had also reported diverse results supporting or opposing the association between *H*. *pylori* infection and CHD [12–14]. In order to answer this important but yet unanswered clinical health issue, a large cohort study such as big data from the Taiwan National Health Insurance Research Database (NHIRD) should be more convincing. Therefore, we aimed to make use of these big data source to analyze and clarify the relevance of *H*. *pylori* eradication and CHD risks.

## Materials and methods

### Ethics statement

This retrospective cohort study was approved by both the institutional review board and the ethics committee of Chang Gung Memorial Hospital and Kaohsiung Medical University Hospital, Taiwan

### Data source

The database used in this study included one million randomly selected patients from the Taiwan NHIRD claims data between the years of 2000 and 2011 which provided coverage for approximately 23 million residents (99% of the population) of Taiwan [15]. We used the inpatient and outpatient claims data as the datasets, and used International Classifications of Diseases, Revision 9, Clinical Modification (ICD-9-CM) to define diseases. All the data calculations in current study were performed by statistician from the center for medical informatics of Kaohsiung Medical Center, Taiwan.

### Study subjects

**Fig 1:**
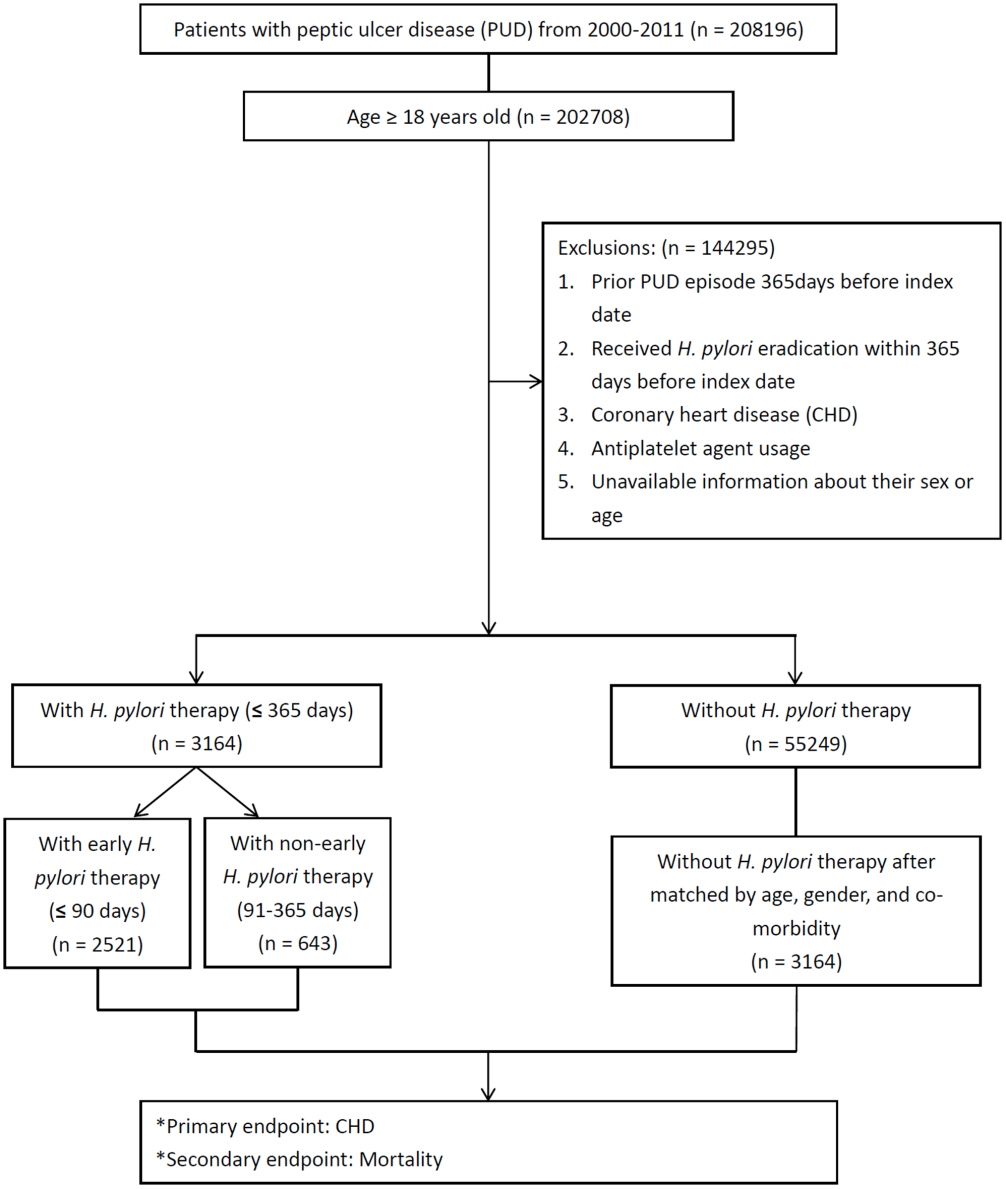
Schematic flowchart of study design.

Fig 1 shows the schematic flowchart of the study design. We enrolled only eligible patients aged more than or equal to 18 years old. We used the date of diagnosis with PUD as index date instead of *H*. *pylori* infection as inclusion criteria because as high as 90% of PUD patients had *H*. *pylori* infection [16]. We identified patients with PUD by using ICD-9-CM codes 531–534 and identified those with CHD by using ICD-9-CM codes 410–414. We identify patients with CHD who had ≥ hospital admission records or ≥ two outpatient visits ≥ 84 days apart. We excluded 144295 patients with *H*. *pylori* eradication within 365 days before the index date, patients who were diagnosed with prior PUD, CHD, antiplatelet agent usage, or without sex or age information.

The patients who received *H*. *pylori* eradication within 365 days of the index date were classified into cohort A (n = 3713). We randomly selected the same number of patients as group A from the non-eradication cohort (n = 55249) to form the comparison group B after matched by age, gender, and Charlson indexed comorbidity using propensity score matching to control potential confounding factors of CHD and all-cause mortality. Comorbid conditions, such as acute myocardial infarction, congestive heart failure, cerebrovascular vascular accident, diabetes mellitus and malignancy had no difference of frequency distribution between groups were excluded from the equation of propensity score.

In this study, we identify patients who received *H*. *pylori* eradication treatment by using drug prescription registry of the NHIRD database when a triple or quadruple therapy consisted of antacid with either a proton-pump inhibitor (PPI) or histamine type 2 receptor antagonists (H_2_RA) in combination with clarithromycin or metronidazole plus amoxicillin or tetracycline prescribed within the same prescription order for 7–14 days. Further subgroup analysis was performed for the time-dependent association between *H*. *pylori* eradication and risk of CHD, interactions between patient demographic characteristics and therapy by age (≥ or < 65 years old). Early *H*. *pylori* eradication was identified in 2521 patients who received treatment ≤ 90 days after the index date.

### Comorbidities, other covariates and outcome+

We assessed general health status by Charlson comorbidity index (CCI), which was a method of predicting mortality by classifying or weighting comorbidities and widely utilized to control for confounding in epidemiological studies [17]. The outcomes of each patient was identified from the NHIRD claims files of CHD patient who had ≥ hospital admission records or ≥ two outpatient visits ≥ 84 days apart.

### Statistical analysis

The number and percentage of patients were calculated for the categorical variables, including age, gender, comorbidities, and medication use. The differences between the two groups were compared by using the chi-square test. Multivariate Cox proportional hazard analysis was used to estimate the hazard ratio (HR) of CHD and mortality, and the 95% confidence interval (CI) among *H*. *pylori* eradication, non-*H*. *pylori* eradication, early *H*. *pylori* eradication and non-early *H*. *pylori* eradication groups. In the models, age, sex, and comorbidities were controlled. To further evaluate the time-dependent association between *H*. *pylori* eradication and risk of CHD, interactions between patient demographic characteristics and therapy were considered and a Cox proportional hazards regression was performed with time dependent covariates in relation to CHD occurrence. Kaplan-Meier curves were also used to display the association of *H*. *pylori* eradication to the occurrence of CHD and mortality over time. The statistical software used in this study was SAS (version 9.4; SAS Institute Inc., Cary, NC, USA). All tests were two-tailed and significance was set at *p* value < 0.05.

## Results

### Demographic data

During the years 2000 to 2011, there were a total of 58413 patients conforming to the inclusion and exclusion criteria. Table 1 demonstrated the demographic characteristics of the study population with and without HP therapy. There were no significant differences in comorbidities in both groups of patients meaning that they were well matched to avoid possible bias during the subsequent analysis.

**Table 1:** Demographic characteristics of the study population with and without HP therapy.

Characteristics	Group A Patients with HP therapy (≤ 365 days) (n = 3164)		Group B Patients without HP therapy (n = 3164)		*P* value
	N	%	N	%	
HP therapy*					
**First**	3141	99.27%	--	--	
HP4+HP3+HP1	3081	98.09%	--	--	
HP4+HP3+HP2	2	0.06%	--	--	
HP5+HP3+HP1	101	3.22%	--	--	
HP5+HP3+HP2	1	0.03%	--	--	
**Second**	24	0.76%	--	--	
HP4+HP6+HP8+HP2	0	0.00%	--	--	
HP5+HP6+HP8+HP2	0	0.00%	--	--	
HP4+HP7+HP1	19	79.17%	--	--	
HP5+HP7+HP1	9	37.50%	--	--	
**Age, years old (mean ± SD)**	47.73±14.24		47.73±14.24		*1*.*0000*
**Age_Class1**					
< 49	1821	57.55%	1821	57.55%	*0*.*9951*
50–59	713	22.53%	713	22.53%	
60–69	353	11.16%	354	11.19%	
≥ 70	277	8.75%	276	8.72%	
**Age_Class2**					
< 65	2741	86.63%	2741	86.63%	*1*.*0000*
≥ 65	423	13.37%	423	13.37%	
**Gender**					
Male	1895	59.89%	1896	59.92%	*0*.*9795*
Female	1269	40.11%	1268	40.08%	
**Charlson score**					
0	2441	77.15%	2441	77.15%	*0*.*9998*
1	649	20.51%	648	20.48%	
2	69	2.18%	70	2.21%	
≥ 3	5	0.16%	5	0.16%	
**Charlson score (mean ± SD)**	0.25±0.49		0.25±0.49		*1*.*0000*
**Charlson comorbidity**					
Dementia	5	0.16%	5	0.16%	*1*.*0000*
Pulmonary disease	95	3.00%	95	3.00%	*1*.*0000*
Connective tissue disorder	14	0.44%	14	0.44%	*1*.*0000*
Peptic ulcer	535	16.91%	535	16.91%	*1*.*0000*
Liver disease	131	4.14%	130	4.11%	*1*.*0000*
Paraplegia	0	0.00%	1	0.03%	*0*.*3173*
Renal disease	11	0.35%	11	0.35%	*1*.*0000*
**Comorbidity**					
Hypertension	286	9.04%	287	9.07%	*0*.*9651*
Hyperlipidemia	115	3.63%	115	3.63%	*1*.*0000*

**Abbreviations**: HP, *Helicobacter pylori*; HIV, human immunodificiency virus

*HP1 = Amoxicillin, HP2 = Metronidazole, HP3 = Clarithromycin, HP4 = PPI, HP5 = H2 blockers, HP6 = Bismuth, HP7 = Levofloxacin, HP8 = Tetracycline

### Outcomes of the study population

The occurrences of CHD and the mortality rate in both cohorts were demonstrated in Table 2. A trend of decreased association of CHD in patients with early eradication compared to those without eradication (2.58% vs. 3.35%, *p = 0*.*0905*). The mortality rate was lower in early eradication subgroup compared to cohort B (2.86% vs. 4.43%, *p = 0*.*0033*). Multivariate analysis showed that hypertension and renal diseases were the risk factors for CHD in patients without eradication whilst younger age (< 65 years old) with *H*. *pylori* therapy was a protective factor (Table 3). Moreover, in those who did not received early eradication, age, male gender and PUD was the risk factors for all-cause mortality (Table 4).

**Table 2:** Outcomes of the study population.

**Characteristics**	**Patients with HP therapy (≤ 365 days) (n = 3164)**		**Patients without HP therapy (n = 3164)**		***P* value**
	**N**	**%**	**N**	**%**	
**Endpoint**					
Coronary heart disease	90	2.84%	106	3.35%	*0*.*2457*
Death	109	3.45%	137	4.33%	*0*.*0686*
Coronary heart disease and death	10	0.32%	18	0.57%	*0*.*1297*
**Characteristics**	**Patients with early HP therapy (≤ 90 days) (n = 3164)**		**Patients without HP therapy (n = 3164)**		***P* value**
	**N**	**%**	**N**	**%**	
**Endpoint**					
Coronary heart disease	65	2.58%	106	3.35%	*0*.*0905*
Death	72	2.86%	137	4.33%	*0*.*0033*
Coronary heart disease and death	4	0.16%	18	0.57%	*0*.*0133*

**Abbreviations**: HP: *Helicobacter pylori*

**Table 3:** Multivariate analysis of potential risk factors for coronary heart disease in patients with peptic ulcer disease (with versus without HP therapy among all ages, by age < and ≥ 65 years old).

Variable	Multivariate analysis			
	HR	95% CI		*P* value
**Group (all ages)**				
Patients without *HP* therapy	1			
Patients with HP therapy (**≤** 365 days)	0.92	0.69	1.22	*0*.*5581*
**Gender (male is reference)**	0.76	0.56	1.03	*0*.*0798*
**Charlson comorbidity**				
Pulmonary disease	1.26	0.66	2.41	*0*.*4795*
Connective tissue disorder	1.76	0.25	12.64	0.5726
Peptic ulcer	0.80	0.55	1.17	*0*.*2496*
Liver disease	0.90	0.44	1.84	*0*.*7655*
Renal disease	7.86	2.88	21.42	*<0*.*0001*
**Comorbidity**				
Hypertension	3.03	2.12	2.25	*0*.*0195*
Hyperlipidemia	1.30	0.69	2.43	*0*.*4196*
**Group (age < 65 years old)**				
Patients without HP therapy	1			
Patients with HP therapy (**≤** 365 days)	0.68	0.46	0.99	*0*.*0464*
**Gender (male is reference)**	0.88	0.59	1.31	*0*.*5170*
**Charlson comorbidity**				
Pulmonary disease	1.31	0.41	4.13	*0*.*6507*
Connective tissue disorder	3.25	0.45	23.43	*0*.*2429*
Peptic ulcer	0.68	0.40	1.15	*0*.*1477*
Liver disease	0.75	0.27	2.06	*0*.*5745*
Renal disease	8.07	1.11	58.50	*0*.*5745*
**Comorbidity**				
Hypertension	2.66	1.49	4.74	*0*.*0009*
Hyperlipidemia	1.70	0.71	4.11	*0*.*2366*
**Group (age ≥ 65 years old)**				
Patients without HP therapy	1			
Patients with HP therapy (**≤** 365 days)	1.4	0.91	2.15	*0*.*1244*
**Gender (male is reference)**	0.55	0.34	0.89	*0*.*0145*
**Charlson comorbidity**				
Pulmonary disease	0.74	0.34	1.65	*0*.*4658*
Peptic ulcer	0.93	0.54	1.60	*0*.*7894*
Liver disease	1.92	0.69	5.36	*0*.*2151*
Renal disease	4.67	1.43	15.19	*0*.*0105*
**Comorbidity**				
Hypertension	1.58	0.98	2.54	*0*.*0585*
Hyperlipidemia	1.21	0.48	3.05	*0*.*6916*

**Abbreviations**: HP: *Helicobacter pylori*; CI: confidence interval

**Table 4:** Multivariate analysis of potential risk factors for mortality in patients with PUD (with and without HP therapy).

Variable	Multivariate analysis			
	HR	95% CI		*P* value
**Group**				
Patients without HP therapy	1			
Patients with HP therapy (**≤** 365 days)	0.86	0.67	1.11	*0*.*2428*
**Age**	1.08	1.07	1.09	*<*.*0001*
**Gender (male is reference)**	0.58	0.44	0.77	*0*.*0001*
**Charlson comorbidity**				
Dementia	2.05	0.50	8.34	*0*.*3178*
Pulmonary disease	1.01	0.59	1.73	*0*.*9610*
Connective tissue disorder	1.19	0.17	8.49	*0*.*8650*
Peptic ulcer	0.69	0.48	0.99	*0*.*0424*
Liver disease	1.07	0.53	2.19	*0*.*8474*
Paraplegia	0	--	--	--
Renal disease	1.78	0.44	7.29	*0*.*4206*
**Comorbidity**				
Hypertension	1.04	0.73	1.48	*0*.*8266*
Hyperlipidemia	0.70	0.32	1.49	*0*.*3518*

**Abbreviations**: HP: *Helicobacter pylori*; CI: confidence interval

### Kaplan-Meier analysis

**Fig 2:**
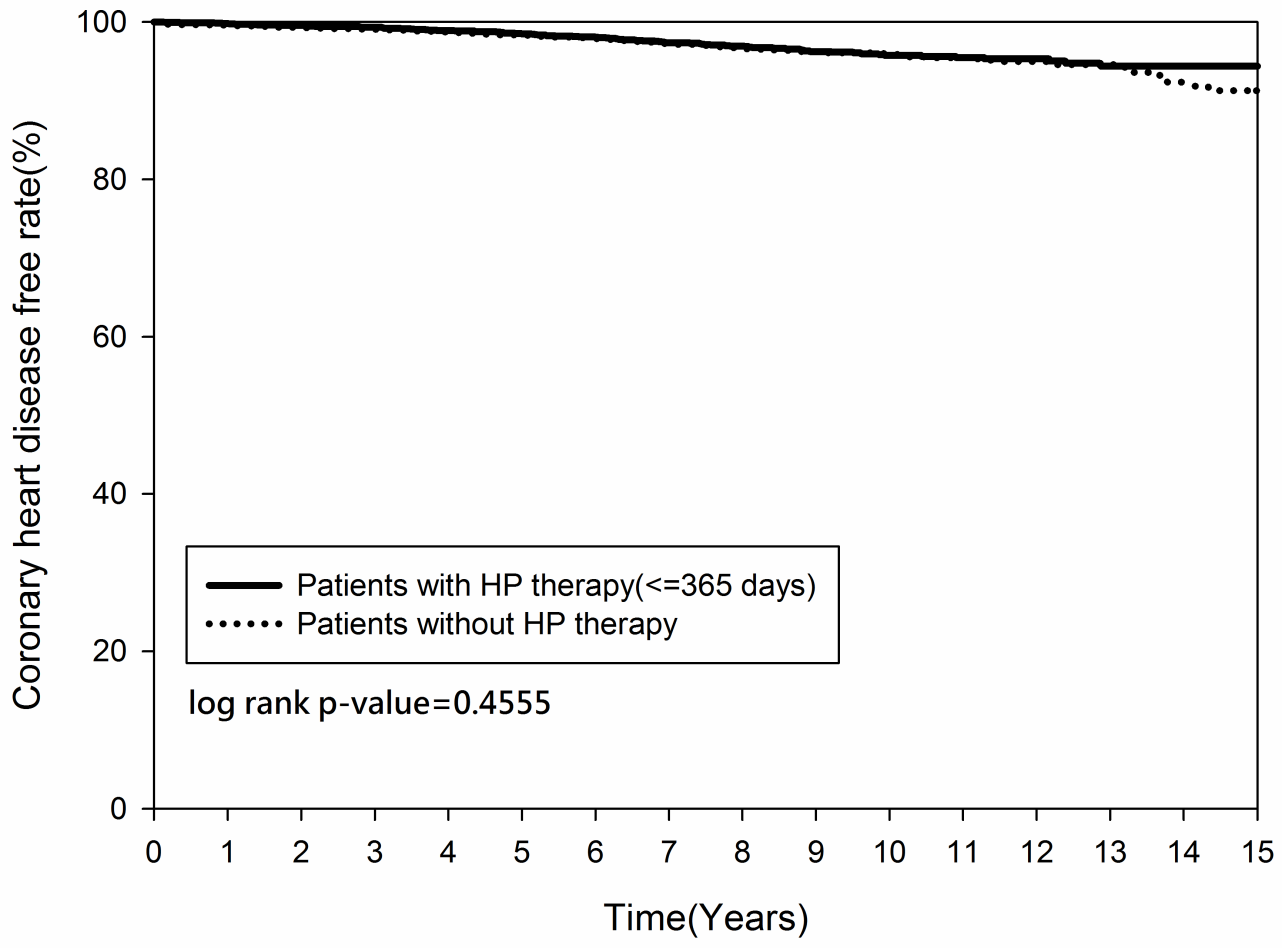
Kaplan-Meier curve for coronary heart disease rate between patients with and without *Helicobacter pylori* therapy.

**Fig 3:**
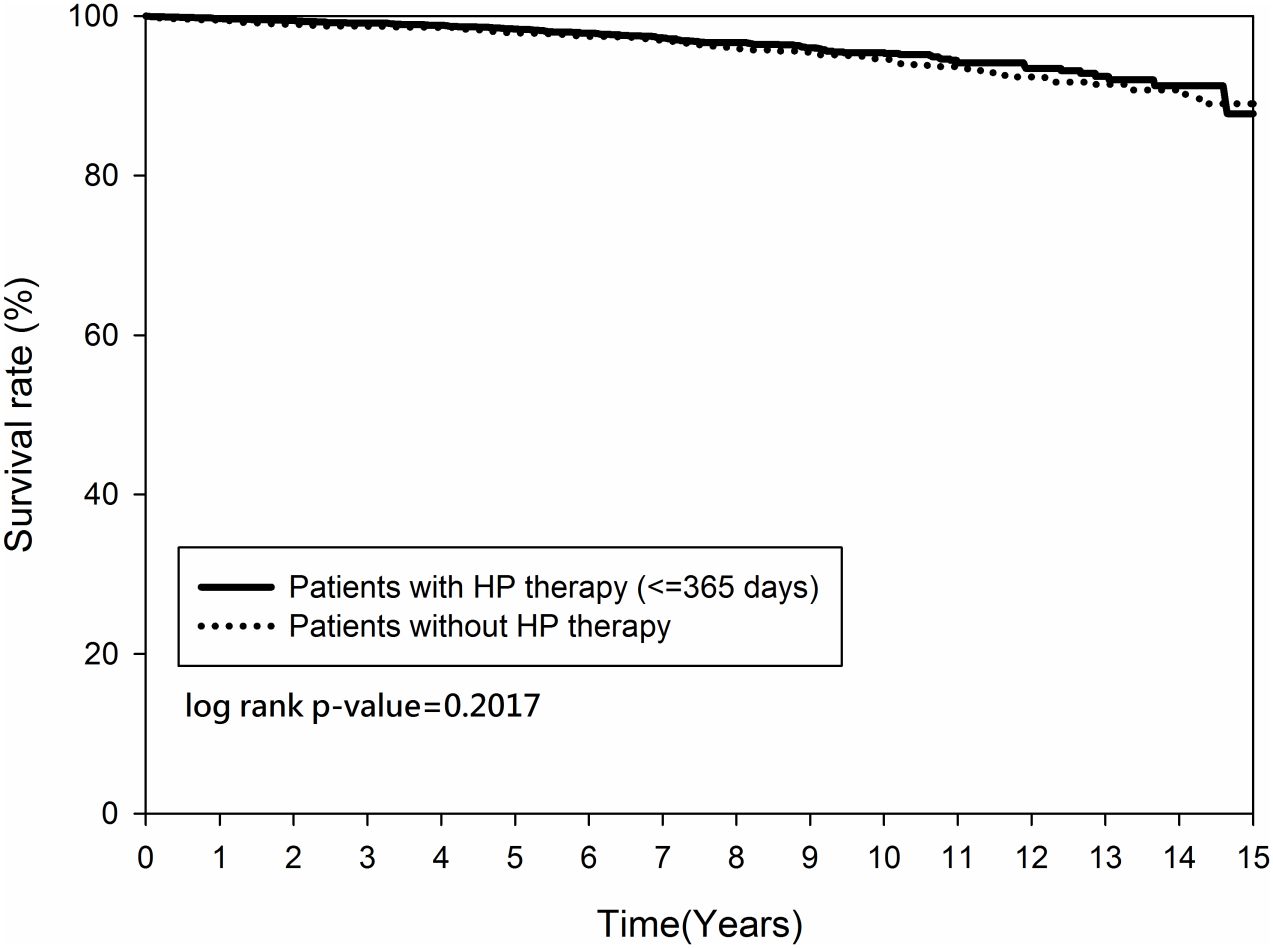
Kaplan-Meier curve for mortality rate between patients with and without *Helicobacter pylori* therapy.

**Fig 4:**
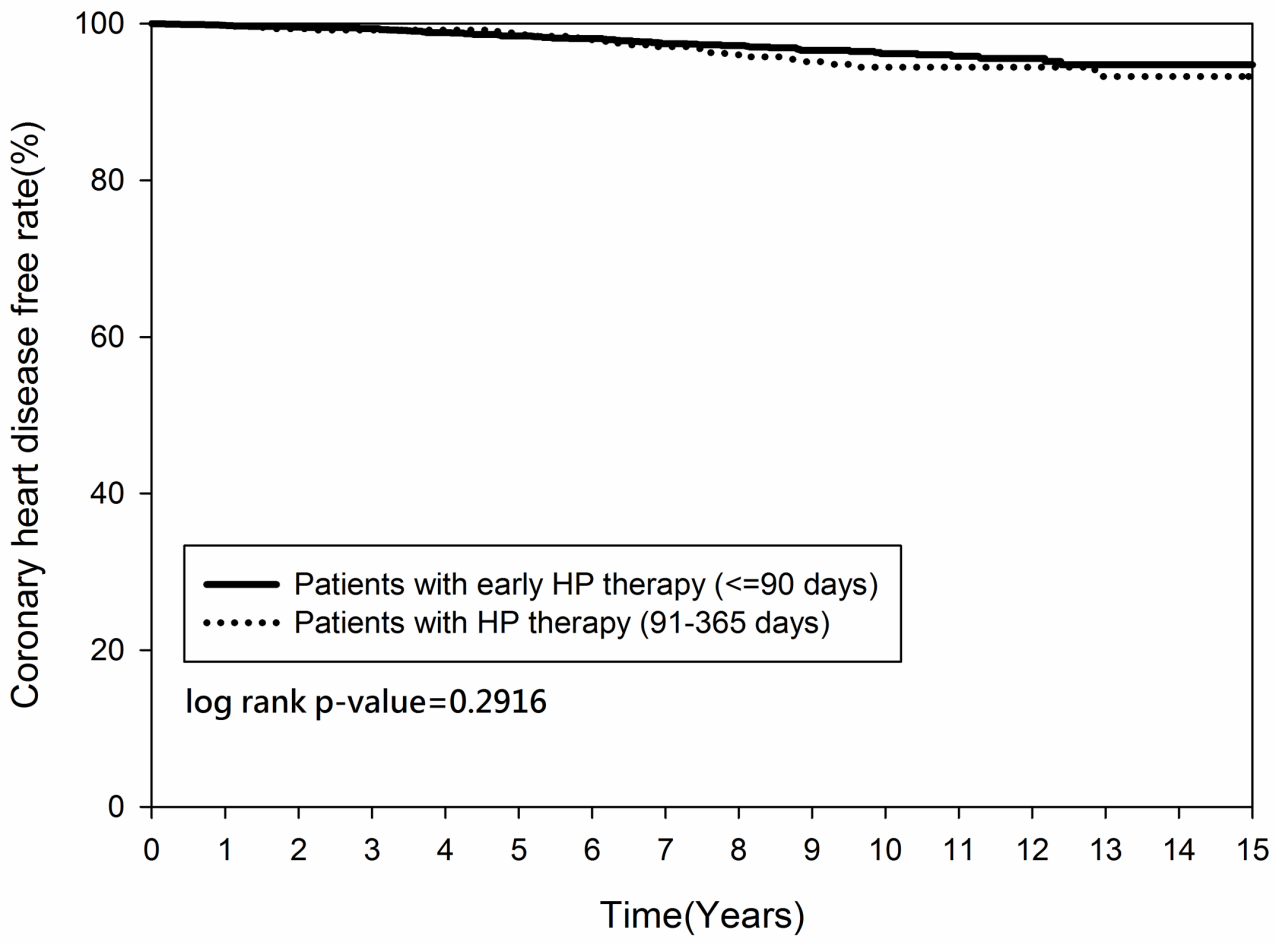
Kaplan-Meier curve for coronary heart disease rate between patients with and without *Helicobacter pylori* therapy, by time of initiation.

**Fig 5:**
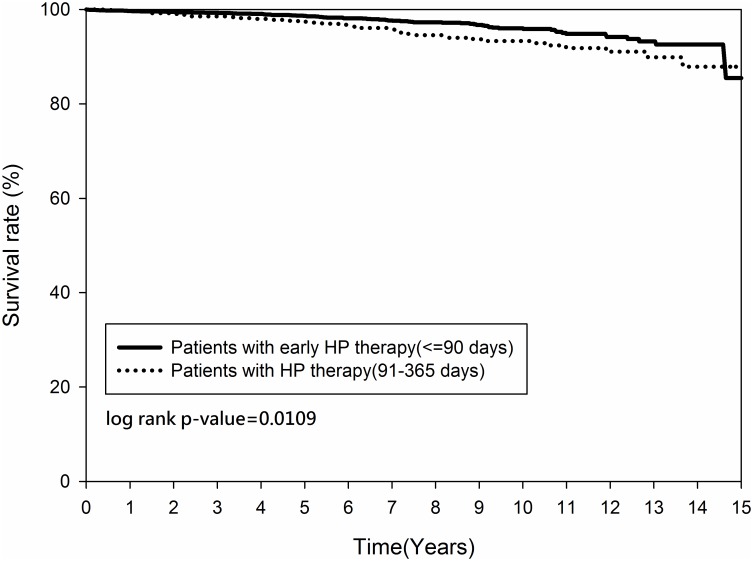
Kaplan-Meier curve for mortality rate between patients with early and non-early *Helicobacter pylori* therapy.

**Fig 6:**
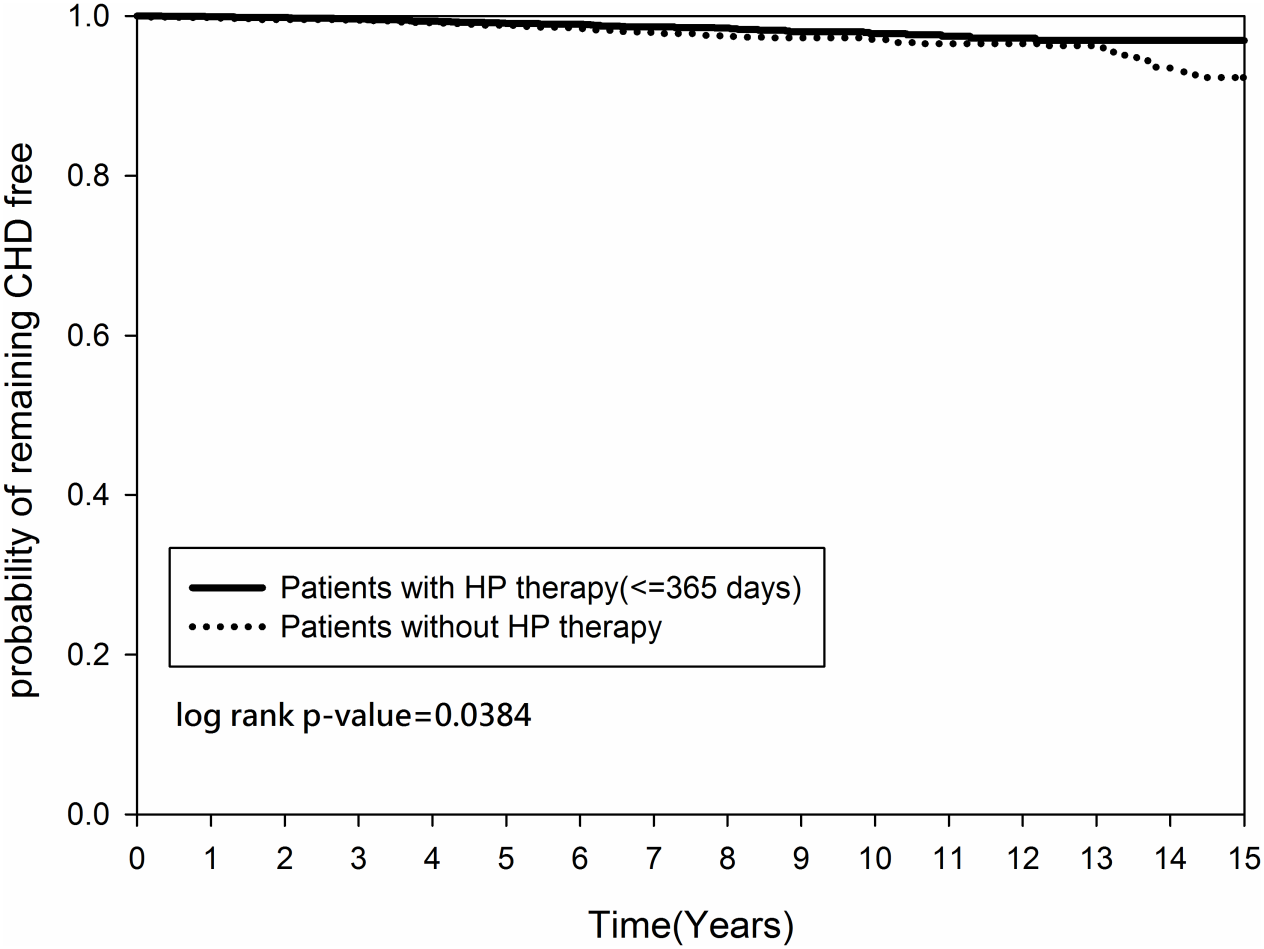
Kaplan-Meier curve for coronary heart disease rate by age between patients (age < 65 years old) with and without *Helicobacter pylori* therapy.

**Fig 7:**
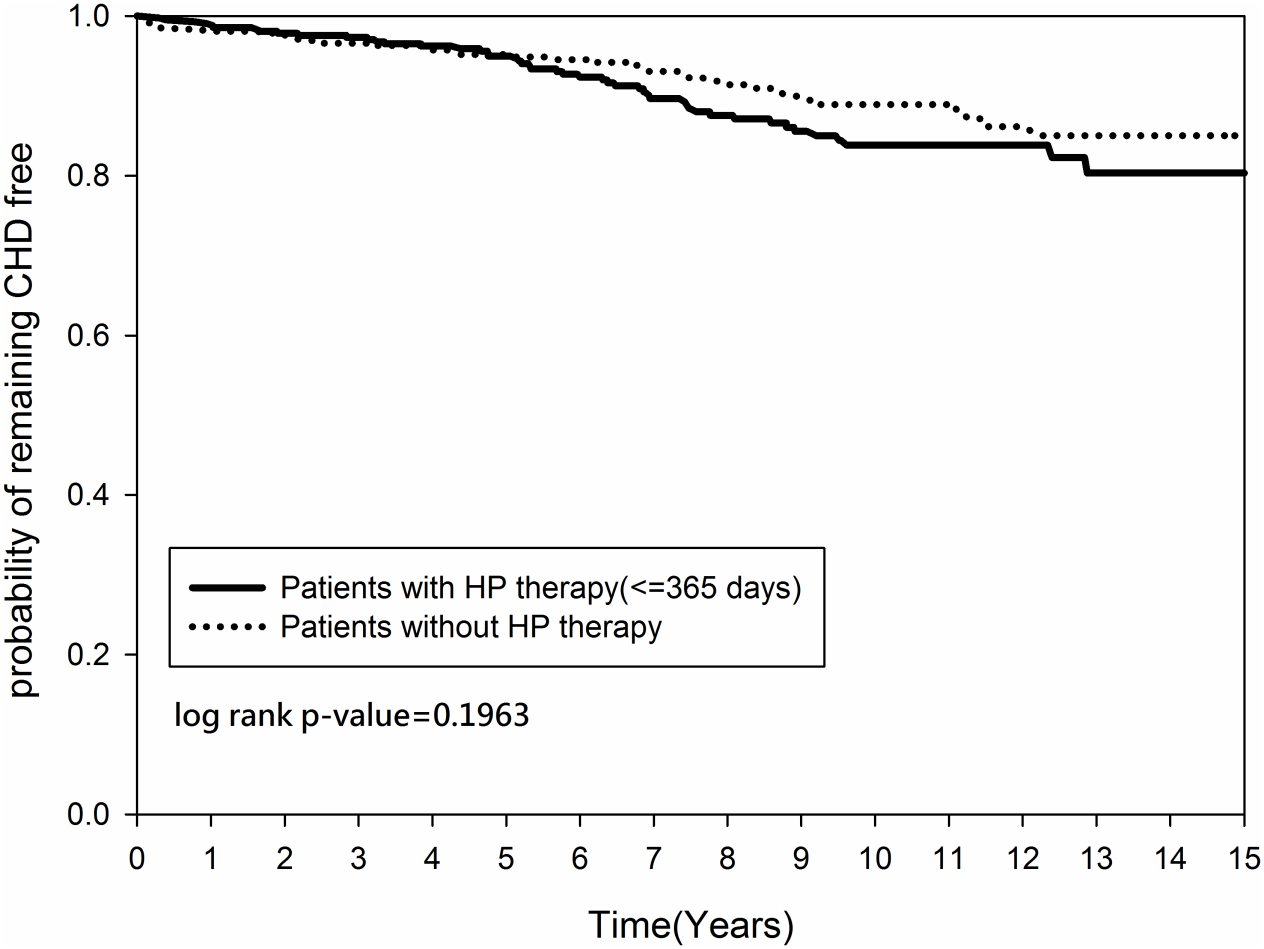
Kaplan-Meier curve for coronary heart disease rate by age between patients (age ≥ 65 years old) with and without *Helicobacter pylori* therapy.

Figs 2 and 3 demonstrated that the cumulative occurrence of CHD and the mortality rate were not significantly different between the two groups since the index date. The cumulative occurrence of CHD between the early and non-early *H*. *pylori* eradication subgroups was similar (*p = 0*.*2916)* (Fig 4) but the mortality rate was higher in the non-early *H*. *pylori* eradication subgroup (*p = 0*.*0109)* (Fig 5). Further, the cumulative CHD rate was significantly lower in younger patients (< 65 years old) with *H*. *pylori* eradication therapy started < 1 year compared to those patients without eradication at all (*p = 0*.*0384)* (Fig 6); the treatment did not appear to have an effect in older patients (≥ 65 years old) (*p = 0*.*1963*) (Fig 7).

## Discussion

The attempt to demonstrate the association between *H*. *pylori* and CHD is always a challenging issue due to the conflicting reports in the literatures. In current study, we used large database and extracted data from Taiwan NHIRD (2000–2011) to clarify the relevance between *H*. *pylori* eradication to CHD in patients with PUD. We observed a trend of decreased association of CHD in patients with early eradication compared to those without eradication and a significant difference observed in composite end-points for CHD and mortality rate in the early eradication subgroup. In addition, the cumulative CHD rate was significantly lower in younger patients younger than 65 years old with *H*. *pylori* eradication therapy started within 365 days compared to those patients without eradication at all.

By searching the literature, these are the evidences we have found. Vafaeimanesh et al. reported that the prevalence of serologically detectable evidence of *H*. *pylori* infection was more in patients with angiographically documented CHD. The evidence of infection was found in more than 70% patients with single vessel disease and double vessel disease but only in 50% individuals without CHD [18]. The other studies also have shown that CHD patients have a higher prevalence of *H*. *pylori* infection [19–21]. However, Danesh et al. conducted a meta-analysis which included 18 epidemiological studies involving 10000 patients but did not find any positive association between *H*. *pylori* and CHD [22].

For those reports which supported *H*. *pylori* eradication could reduce the risk of CHD, it was believed that the timing of eradication mattered. Nozaki et al. found that *H*. *pylori* eradication at an early stage of inflammation (< 15 weeks) might be effective in preventing gastric carcinogenesis [23]. Kowalski et al. found that the patients with serological evidence of *H*. *pylori* infection had the higher loss of coronary lumen, and compared with the placebo group, eradication of *H*. *pylori* attenuated this reduction in lumen of the coronary artery [24]. However, there is by far no other study to further assess the long-term effect of *H*. *pylori* eradication on the incidence of new CHD. This could account for the results in our study that there was significantly lower cumulative CHD rate in patients younger than 65 years with *H*. *pylori* eradication therapy started within 365 days and mortality rate in the early eradication subgroup at the long-term follow-up.

The possible direct and indirect mechanisms of *H*. *pylori* related CHD included induction of inflammatory response secondary to chronic infectious state, endothelial damage, chronic low grade activation of coagulation cascade, dysregulation of lipid metabolism, and hyperhomocysteinaemia [25]. Another larger study showed that the eradication of *H*. *pylori* seemed to increase HDL levels and reduce the levels of C reactive protein (CRP) and those of fibrinogen [26]. Gen et al. demonstrated changes in lipid profile including an increase in HDL levels and a fall in low density lipoprotein (LDL) levels with *H*. *pylori* eradication [27]. Corrado et al. found that chronic *H*. *pylori* infection induces increase of level of the gastric juice and decrease of ascorbic acid levels, both of which cause folate absorption reduction. Low folate hampers the methionine synthase reaction, and it will increase blood hemocysteine concentration which results in the damage of the endothelial cells [28]. Therefore, we believe that early *H*. *pylori* eradication could decrease CHD risks especially in those aged < 65 years.

Our study has certain strengths. First, this was a big data study with large sample size from Taiwan NHIRD database which was a nationwide cohort. Second, the important confounding variables for CHD and mortality were available in detail from NHIRD, and were excluded to reduce the confounding effects. Importantly, as shown in Table, we successfully matched the two groups as there were no significant differences in comorbidities in both groups of patients to avoid possible bias during the subsequent analysis.

However, there are still some limitations in our study. First, several published meta-analysis studies reported positive association between cytotoxin-associated protein (Cag-A) positive strain of *H*. *pylori* infection and CHD [29, 30], but we couldn’t define Cag-A positive or Cag-A negative strain of *H*. *pylori* infection by ICD-9-CM codes. Second, the patient numbers of composite end points for CHD and mortality are rather small, which may have relatively low power in statistical analysis. Third, we were unable to evaluate the patients’ socio-economic disparities which could be associated to both CHD and *H*. *pylori* infection as these data were unavailable in the NHIRD database. Common limitations of the claims data include lack of information on body mass index, level of glucose and lifestyle, which could affect the interpretation of the present study. Finally, as high as > 90% of *H*. *pylori* were found in patients with duodenal ulcers and 70–90% in gastric ulcer patients [31]. In addition, true *H*. *pylori* infection may be underdiagnosed among patients with peptic ulcer patients. It is expected that a high-level of significant association between *H*. *pylori* eradication and CHD will be considered if more true *H*. *pylori* infections were identified in practice settings.

In conclusion, the trend of decrease in CHD occurrence after early *H*. *pylori* eradication in addition to the significant decrease in composite end points for CHD and death, the significantly lower cumulative CHD rate in younger patients < 65 years old with *H*. *pylori* treated within 365 days suggested that there was positive association between *H*. *pylori* eradication and CHD.

## Supporting information

S1 File S1 HP-CHD PLOSONE.xls. HP365 by age. This file provides data of the multivariate analysis of potential risk factors for coronary heart disease in patients with peptic ulcer disease (with versus without *H*. *pylori* therapy among all ages, by age < and ≥ 65 years old) in manuscript.
(XLS)

## Abbreviations

CCI: Charlson comorbidity index
CHD: coronary heart disease
CI: confidence interval
*H. pylori*: *Helicobacter pylori*
H_2_RA: histamin-2 receptor antagonists
HIV: human immunodificiency virus
HR: hazard ratio
ICD-9-CM: International Classifications of Diseases, Revision 9, Clinical Modification
NHI: National Health Insurance
NHIRD: National Health Research Institute database
PPI: proton pump inhibitors
PUD: peptic ulcer diseases
SD: standard deviation
